# Determination of Decabrominated Diphenyl Ether in Soils by Soxhlet Extraction and High Performance Liquid Chromatography

**DOI:** 10.1155/2013/840376

**Published:** 2013-11-05

**Authors:** Xing-Jian Yang, Zhi Dang, Fang-Li Zhang, Zhao-Ying Lin, Meng-Yao Zou, Xue-Qin Tao, Gui-Ning Lu

**Affiliations:** ^1^School of Environment and Energy, South China University of Technology, Guangzhou 510006, China; ^2^The Key Lab of Pollution Control and Ecosystem Restoration in Industry Clusters, Ministry of Education, Guangzhou 510006, China; ^3^School of Environmental Science and Engineering, Zhongkai University of Agriculture and Engineering, Guangzhou 510225, China

## Abstract

This study described the development of a method based on soxhlet extraction combining high performance liquid chromatography (soxhlet-HPLC) for the accurate detection of BDE-209 in soils. The solvent effect of working standard solutions in HPLC was discussed. Results showed that 1 : 1 of methanol and acetone was the optimal condition which could totally dissolve the BDE-209 in environmental samples and avoid the decrease of the peak area and the peak deformation difference of BDE-209 in HPLC. The preliminary experiment was conducted on the configured grassland (1 **μ**g/g) to validate the method feasibility. The method produced reliable reproducibility, simulated soils (*n* = 4) RSD 1.0%, and was further verified by the analysis e-waste contaminated soils, RSD range 5.9–11.4%. The contamination level of BDE-209 in burning site was consistent with the previous study of Longtang town but lower than Guiyu town, and higher concentration of BDE-209 in paddy field mainly resulted from the long-standing disassembling area nearby. This accurate and fast method was successfully developed to extract and analyze BDE-209 in soil samples, showing its potential use for replacing GC to determinate BDE-209 in soil samples.

## 1. Introduction

Polybrominated diphenyl ethers (PBDEs) are widely used brominated flame retardants which prevent ignition in textiles, plastics, and electronic products [1–3]. The worldwide commercial produced PBDEs mainly consist of 80% decabrominated diphenyl ether (BDE-209) with minor octabrominated diphenyl ethers (Oct-BDE) and pentabrominated diphenyl ethers (Penta-BDE) [4]. It is estimated that 40–70% of the global e-waste is imported to China [5, 6]. Due to the “nonstandard” disposal of the e-waste such as open incineration and family-type manual dismantling, heavy metals, PBDEs, and polychlorinated biphenyls (PCBs) are being released to the environment, making it a potential risk to the water, air, and soil around the e-waste disposal site [7].

The e-waste pollution has been an important environmental problem in the e-waste disposal areas. Due to the wide use of BDE-209, it appears normal that BDE-209 is a predominant congener in the environment [8–10]. At burning site in Longtang town of South China, BDE-209 soil concentration ranged 66.7–284 ng/g with an average of 46.2 ng/g [11]. BDE-209 has lower toxicity among the congeners, but it has the risk of debromination forming toxic intermediates. Several studies were made on the debromination pathways and intermediate products of BDE-209 [12–17]. It was indicated that BDE-209 could form lower brominated and more toxic congeners through photolytic debromination, anaerobic biodegradation, and biota metabolism.

Since BDE-209 is used as the most addictive chemicals, with properties of easy thermolysis and debromination, it is necessary that an accurate and fast method be developed to determine the concentration in environmental matrices especially soils. Although quantification of BDE-209 by gas chromatography coupled with mass spectrometer (GC-MS) has been widely reported [18–21], the thermolysis of BDE-209 in the capillary column is difficult to be avoided. Compared to the technique of GC-MS, few studies have reported the determination of the BDE-209 by HPLC. The concentration of BDE-209 in water, urine, plastics, and simulated soils samples is easy to be quantified compared to e-waste dismantling site soils which have more interfering substances and capacity of retaining BDE-209 (log⁡*k*_ow_ = 10) through physical or chemical adsorption [13, 22–25]. A factor should also be considered that BDE-209 is a nonpolar substance and it can dissolve in nonpolar solvents such as hexane, toluene, or acetone. The previous study indicated that BDE-209 can not completely solubilize in methanol and acetonitrile which are recommended mobile phases [23]. It was proved by facts that the solvent must totally dissolve in the mobile phase, so hexane perhaps is not a good choice. Thus, the influence of solvent effect must be considered.

In the present study, conventional soxhlet extraction combined with HPLC was used to analyze BDE-209 in soils. The aims of this study were (1) to develop an accurate and fast method for determination of the BDE-209 in soils, (2) to investigate the influence of the solvent effect, and (3) to compare the BDE-209 contamination level with other regions.

## 2. Experimental Section

### 2.1. Reagents and Materials

BDE-209 (99.0% purity) was purchased from Dr. Ehrenstorfer (Augsburg, Germany). Acetone and dichloromethane were purchased from J. T. Baker (center valley, USA) and hexane was from Fisher (USA). The HPLC-grade methanol was purchased from CMW, Shanghai, China. The stock standard solution of BDE-209 was made by dissolving BDE-209 in acetone (20 *μ*g/mL), which differs from the previous study [23]. The working standard solutions were prepared by series dilution of stock standard solutions with methanol. Deionized water was purified with Millipore Mill-Q plus system.

### 2.2. Sample Collection and Preparation

For verifying feasibility of the method, the soils were sampled in three different sites which are, respectively, located in Guiyu town, Longtang town and grassland near the campus of South China University of Technology. Guiyu town and Longtang town are located in Shantou city and Qingyuan city, Guangdong Province, China, respectively. These towns contain large informal e-waste recycling centers [26, 27]. The grassland, which is located near the campus of South China University of Technology, was uncontaminated. 

The sampling locations can be classified into four groups: burning site near the disassemble workshop in Guiyu (GBS), paddy field near the disassemble workshop in Guiyu (GPF), burning site in Longtang (LBS), and grassland near the campus (CGL). All samples were collected from top soil (0–10 cm) and packed in polythene bags. The samples were freeze-dried, sieved to 60 mesh, and stored at −20°C prior to analysis. Grassland soil was spiked with 1 *μ*g/g of BDE-209.

### 2.3. Solvent Effect Experiment

A series of experiments were conducted to observe the solvent effect of working standard solutions in HPLC. A 20 *μ*g/mL BDE-209 stock standard solution was prepared and diluted by a different ratio of acetone and methanol. The working standard solutions were analyzed by HPLC and the effect of acetone ratio on the peak area of BDE-209 was observed.

### 2.4. Soil Extraction, Cleanup, and Analysis

Briefly, 8 g freeze-dried soil samples was weighed and soxhlet extracted with acetone and hexane (1 : 1, v : v, sic passim) for 48 h at 60°C with active copper (3 g) was added to remove the sulfur. The extracts were concentrated to 1-2 mL. The residue acetone, which tends to influence the performance of chromatographic column, was removed through solvent-exchange to hexane. The soil extracts were concentrated to 1ml and cleaned up using 1 or 2 chromatography columns (10 mm, diameter) packed with neutral alumina (6 cm), neutral silica gel (2 cm), alkaline silica gel (5 cm), neutral silica gel (2 cm), and sulfuric acid silica gel (8 cm) from the bottom to the top. A total of 60 mL of 1 : 1 hexane and dichloromethane was used as the eluent. The extracts were evaporated until almost dryness under a gentle stream of nitrogen and reconstituted with acetone and methanol (1 : 1) to a final volume of 1 mL.

The cleaned-up extracts were analyzed by Agilent 1200 HPLC equipped with a manual injector and diode array detector (DAD). A Luna-PFP2 column (250 mm × 4.6 mm, 5 *μ*m particle size), with excellent selectivity to aromatic compounds, was used. 20 uL of extracts was manually injected. The operating conditions were mobile phase, methanol water = 95 : 5 (v : v); flow rate, 1 mL/min; column temperature, 30 ± 1°C; the wavelength of detector, 226 nm. The retention time of BDE-209 was 9.8 ± 0.1 min with the above conditions. Procedural blanks, spiked blanks, and recovery tests were conducted to evaluate contamination and interference in experiment process. 

## 3. Results and Discussion

### 3.1. Solvent Effect on the Peak Area of Working Standard Solutions

**Figure 1:**
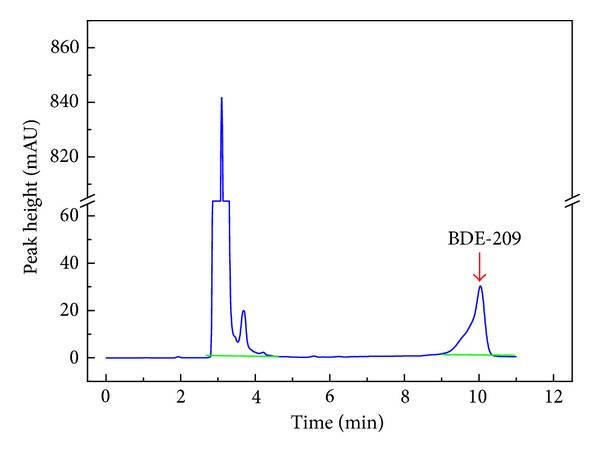
The peak deformation difference of BDE-209.

Due to the incomplete dissolution of BDE-209 in methanol and acetonitrile, other solvents should be explored. Acetone was considered in this study, which was totally miscible with methanol and water. The results showed that correlation coefficient (*r* ^2^) only reached 0.995, which is similar to acetonitrile-made standard curves [23]. The single solvent also caused peak deformation difference (Figure 1). This was mainly caused by high acetone concentration that was not match with the mobile phase. 

**Figure 2:**
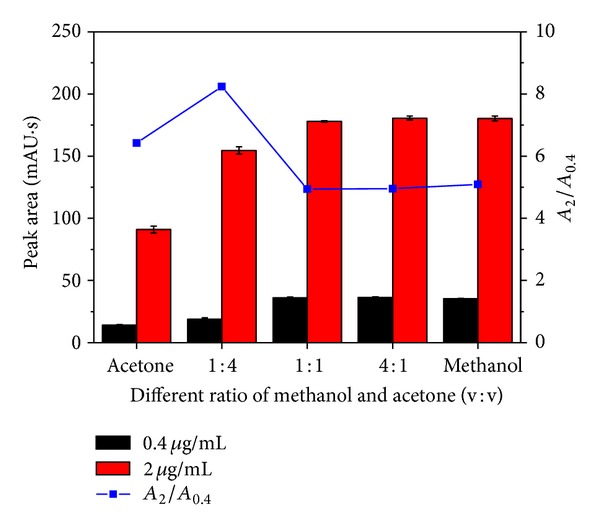
Different ratios of methanol and acetone on peak area of BDE-209 (blue line represents the peak area ratio of 2 *μ*g/mL and 0.4 *μ*g/mL solutions (*A*_2_/*A*_0.4_) at various amount of acetone).

**Figure 3:**
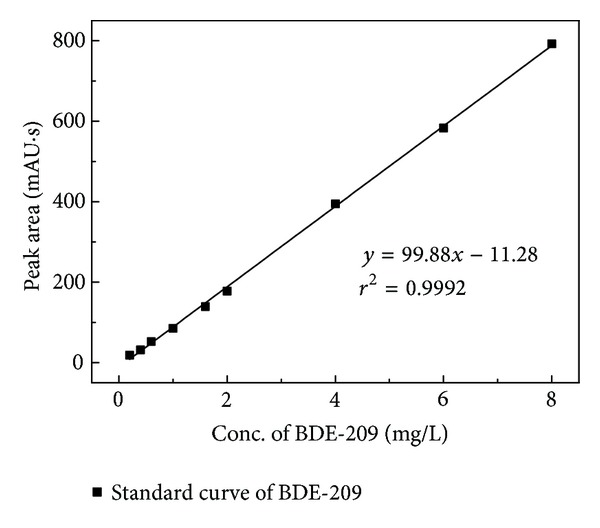
Standard curve of BDE-209.

Thus, another experiment was conducted on dilution of stock standard solutions by a different ratio of acetone and methanol. The BDE-209 concentrations around e-waste sites are particularly high; thus different concentrations of working standard solutions were considered. As shown in Figure 2, the peak areas had no substantial changes when the ratio ranged from 1 : 1 to 4 : 1. But when the ratio was 1 : 4 and 1 : 0, the peak area started to decrease rapidly. The statistical analysis (SPSS) indicated that the 2 sets of data had a significant difference compared to other sets (*P* < 0.05). This showed that more acetone will lead to a decrease in peak area of BDE-209 and in turn influence the accuracy of BDE-209 concentration. Acetone has a strong ultraviolet adsorption under 330 nm, it is a kind of strong solvent which has more powerful elution force than methanol and acetonitrile. It takes short time for sample solutions to reach the chromatographic column and the sample solutions would not be fully diluted by mobile phase. The acetone will exist locally, leading to the elution velocity increase. So, it is nothing exceptional that the concentration and shape of peak area will be irregular [28]. The peak area ratio of 2 *μ*g/mL and 0.4 *μ*g/mL solutions (*A*_2_/*A*_0.4_) was calculated by the mean values of peak areas in a different ratio of acetone and methanol. The results (Figure 2) showed that *A*_2_/*A*_0.4_ for 1 : 4 and 1 : 0 solutions were 8.2 and 6.4, respectively, while others were close to 5.0, equal to the concentration proportion of BDE-209. This could explain the inaccuracy of correlation coefficient (*r* ^2^) when acetone made up more proportion. With the limited solubility of BDE-209 in methanol, it is necessary that the mixed solutions can totally dissolve the BDE-209 in environmental samples. Thus, a ratio of 1 : 1 (acetone and methanol) was selected for optimal combination, with a *r* ^2^ of 0.9992 (Figure 3), for BDE-209 concentration range 0.2 to 8 *μ*g/mL. 

### 3.2. Verification of Method Feasibility

In order to investigate the performance of the proposed method, the soil samples from grassland were analyzed under the above conditions. The soils were analyzed in four parallel samples. The results (Table 1) showed good repeatability with a RSD of 1.0%. Due to the high concentration of BDE-209 that was detected in the simulated soil, it indicated that 1 : 1 of acetone and methanol can completely satisfy the analysis of environmental soil samples.

As the feasibility of the method in the analysis of contaminated simulated soils, this research was further concentrated on verification of method feasibility on real environmental soil samples (Table 1). The RSD was 11.4%, 8.9%, and 5.9% for QBS, GBS, and GPF, respectively. E-waste contaminated soils are usually more complex which contain a large number of PBDEs, PCBs, PAHs, and heavy metals, leading to measurement difficulties. Results of QBS, GBS, and GPF indicated that the developed method was feasible. 

**Figure 4:**
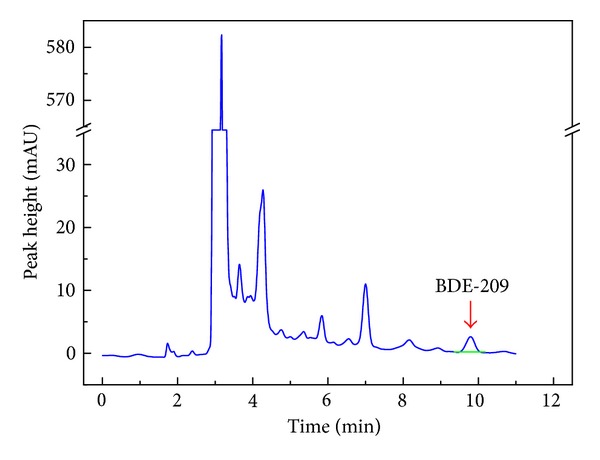
The peak of BDE-209 in QBS.

The factor of impurity interference should be considered during analysis. White precipitate was observed in chromatography bottle when it was reconstituted with acetone and methanol. There was no report on this phenomenon when it was reconstituted with hexane [32, 33]. The pretreatment was a crucial step for accurate quantification of BDE-209; more soil samples extracted with solvents will inevitably lead to an increase in impurities. The polarity of the solvent system was enhanced when methanol was added to the simple system of acetone, which leads to the white solid precipitation. This phenomenon could be avoided by some preliminary judgment: (1) the color of extracts in the top of column when it has reacted with acidic silica gel; (2) flow rate of eluting solutions in columns; (3) whether the effluent in the column was colorless transparence; (4) whether the color of solutions tended to change when the effluent was transferred to chromatography bottle by hexane and dichloromethane. If the color of extracts was deep, the flow rate decreased, the effluent was not transparent, or color of the solution tended to change, it was indicated that soil samples existed had lots of impurities. Thus, 2 or more chromatographic columns were needed to further clean up the soils. In our study, more columns were used if needed, and the solutions were transparent when reconstituted with acetone and methanol. The HPLC graph showed that the peak of BDE-209 could avoid other interference of organic compounds such as PBDEs and PCBs (Figure 4).

### 3.3. Quality Assurance and Quality Control

Procedural and spiked blanks were processed for evaluating interferences, contamination, and the accuracy of the experiment process. A recovery test was also performed by adding BDE-209 to the treated soil samples.  Table 2 showed good recovery for the tests. Recovery ranged from 91.1 to 116.1%, and spiked blanks recovery ranged from 91.8 to 102.0%, which satisfied the range required (80–120%). The BDE-209 concentration was below detection in the procedural blanks.

### 3.4. Comparison in Levels of BDE-209

High level of BDE-209 was observed in paddy field in Guiyu (368.1 ng/g).  Table 3 exhibited the BDE-209 concentration in this study and other studies. The concentration of BDE-209 in our study was supposed to be the highest. The result probably resulted from multiple factors. Plants usually have capacity to absorb BDE-209 by roots from soils and uptake it to stem and leaves. However, lower brominated PBDEs are more liable to be taken up by plants than BDE-209 [34, 35]. BDE-209 was also tended to be photodegradated in natural conditions [13, 14]. Soil is a receptor for both particulate and gaseous atmospheric pollutants. Highly brominated congeners tend to affiliate with particles, so they tend to be scavenged from the air mass than low brominated congeners, and they preferentially deposit in the nearby farmland soils [30]. Above all, the sampling paddy field was short distance from the long-standing disassembling area, which predominantly contributed to the high level of BDE-209 in paddy field. The BDE-209 accumulation rate in soils was larger than the removal rate from soils by plants and photodegradation. 

The BDE-209 concentration in burning sites of Longtang and Guiyu towns was 53.6 ng/g and 132.1 ng/g, respectively, which was mainly contributed by combusted residues and ash of e-waste. The BDE-209 concentrations were almost the same as the e-waste sites in previous study of Longtang towns, but were lower than the concentration in Guiyu town. 

## 4. Conclusions

The solvent effect of acetone and methanol was investigated for accurate quantification of BDE-209. 1 : 1 of acetone and methanol was identified as optimal solvent ratio, which had a high performance on solubilization of BDE-209 in soil samples and could avoid the interference of acetone peak under 330 nm. The concentration of BDE-209 from paddy field was 368.1 ng/g, exhibiting a predominantly contribution by long-standing disassembling area. The BDE-209 concentration in burning sites of Longtang and Guiyu towns was 53.6 ng/g and 132.1 ng/g, respectively. The investigated different soil samples showed no matrix interference on the proposed method (recovery 91.1–116.1%). This developed method can be extensively used in analyzing the residue BDE-209 in environmental soil samples.

## Figures and Tables

**Table 1:** Concentration of BDE-209 in different sites.

Sampling site	Mean concentration (ng/g dry wt.)	RSD%
CGL^1^ (*n* = 4)	999.3	1.0
QBS (*n* = 5)	53.6	11.4
GBS (*n* = 5)	132.1	8.9
GPF (*n* = 3)	368.1	5.9

^1^Simulated soils manual contaminated with 1 ug/g BDE-209.

**Table 2:** Procedural, spiked blanks and recovery test of soil samples.

Sampling site	Procedural blank %	Spiked blank %	Recovery test %
QBS	nd	102.0	116.1
GPF	nd	91.8	113.8
CGL	nd	99.7	91.1

nd: not detected.

**Table 3:** Comparison of BDE-209 concentrations with other studies.

Type	Region	BDE-209 (ng/g)	Reference
Farmland	Paddy field, Guiyu	368.1	This study
	Rice field, Guiyu	37.3	[8]
	Farmland, Qingyuan	32.2 (3.5–178.5)	[29]
	Farmland near open burning site, Qingyuan	29.9 (1.3–182.7)	[29]
	Paddy field, Qingyuan	12.4 (8.0–18.7)	[11]
	Farmland, Guiyu	31.6	[26]
	Farmland, Qingyuan	41.9	[26]

Burning site	Burning site, Qingyuan	53.6	This study
	Burning site, Guiyu	132.1	This study
	Burning site, Qingyuan	154 (66.7–284)	[11]
	Open burning site, Qingyuan	693.3	[30]
	Open burning site, Guiyu	630	[31]
